# Molecular Insights in the Anticancer Activity of Natural Tocotrienols: Targeting Mitochondrial Metabolism and Cellular Redox Homeostasis

**DOI:** 10.3390/antiox14010115

**Published:** 2025-01-20

**Authors:** Raffaella Chiaramonte, Giulia Sauro, Domenica Giannandrea, Patrizia Limonta, Lavinia Casati

**Affiliations:** 1 Department of Health Sciences, Università degli Studi di Milano, 20142 Milan, Italy; raffaella.chiaramonte@unimi.it (R.C.); giulia.sauro@unimi.it (G.S.); domenica.giannandrea@unimi.it (D.G.); 2 Department of Pharmacological and Biomolecular Sciences “R. Paoletti”, Università degli Studi di Milano, 20133 Milan, Italy; patrizia.limonta@unimi.it

**Keywords:** cancer cells, mitochondrial metabolic reprogramming, ROS generation, redox homeostasis, tocotrienols, anticancer activity, novel anticancer strategies

## Abstract

The role of mitochondria as the electric engine of cells is well established. Over the past two decades, accumulating evidence has pointed out that, despite the presence of a highly active glycolytic pathway (Warburg effect), a functional and even upregulated mitochondrial respiration occurs in cancer cells to meet the need of high energy and the biosynthetic demand to sustain their anabolic growth. Mitochondria are also the primary source of intracellular ROS. Cancer cells maintain moderate levels of ROS to promote tumorigenesis, metastasis, and drug resistance; indeed, once the cytotoxicity threshold is exceeded, ROS trigger oxidative damage, ultimately leading to cell death. Based on this, mitochondrial metabolic functions and ROS generation are considered attractive targets of synthetic and natural anticancer compounds. Tocotrienols (TTs), specifically the δ- and γ-TT isoforms, are vitamin E-derived biomolecules widely shown to possess striking anticancer properties since they regulate several intracellular molecular pathways. Herein, we provide for the first time an overview of the mitochondrial metabolic reprogramming and redox homeostasis perturbation occurring in cancer cells, highlighting their involvement in the anticancer properties of TTs. This evidence sheds light on the use of these natural compounds as a promising preventive or therapeutic approach for novel anticancer strategies.

## 1. Introduction

Mitochondria, referred to as the “powerhouses of the cell”, play a prominent role in aerobic respiration and energy generation through oxidative phosphorylation (OXPHOS). In the inner membrane of mitochondria, coenzymes NADH and FADH_2_, mainly formed through the tricarboxylic acid (TCA) cycle, donate electrons and protons to the electron transport chain (ETC). Electrons are then transferred to O_2_, while protons are pumped into the mitochondrial inter-membrane space, creating a proton gradient used for ATP synthesis [1].

Dysregulated cellular metabolism has emerged as a hallmark of tumors, with cancer cells reprogramming mitochondrial function metabolism to meet the higher demands for energy and biosynthetic precursors required for rapid cell proliferation, survival, and metastasis. This metabolic reprogramming is now recognized as a critical feature of cancers [2,3,4,5,6,7,8,9]. Mitochondria are also the primary source of reactive oxygen species (ROS) generated as byproducts of ETC activity [10,11]. The balance of ROS levels, primarily generated from the leakage of the ETC due to oxidative phosphorylation dysfunction, is crucial for mitochondrial function, redox homeostasis, regulation of cell proliferation and differentiation, and intracellular Ca^2+^ homeostasis [12,13]. In normal cells, low ROS levels act as signaling molecules to promote cell proliferation and survival. On the other hand, cancer cells, characterized by moderate ROS levels, protect themselves by enhancing antioxidant defenses to maintain ROS at levels that promote tumor initiation, progression, and metastasis [14,15]. However, excessive ROS beyond the cytotoxic threshold dysregulate redox signaling and cause oxidative damage, leading to cancer cell death [16,17,18,19,20].

Building on these observations, impairing mitochondrial functions and triggering ROS formation are emerging as promising therapeutic approaches for anticancer treatment [21,22,23,24,25,26,27,28,29,30,31].

Among the compounds affecting mitochondrial functions, vitamin E is one of the most prominent antioxidants. It is a fat-soluble vitamin comprising eight compounds: four tocotrienols (α-, β-, δ-, and γ-TTs) and four tocopherols (TPs). TTs are now recognized for their superior antioxidant and anti-inflammatory properties compared to TPs, attributed to their more efficient serum protein-mediated cellular uptake [32,33]. Notably, δ- and γ-TT have demonstrated significant anticancer properties, inducing growth arrest and cell death in several cancer cell types by activating diverse signaling pathways [34,35,36,37,38,39].

This review provides an overview of mitochondrial metabolic reprogramming and redox homeostasis perturbation in cancer growth and progression, with a detailed overview of recent advances in understanding the anticancer mechanisms of TTs.

## 2. Mitochondrial Metabolic Reprogramming

Dysregulation of cellular metabolism has recently emerged as a hallmark of cancer. Cancer cells require large amounts of metabolites and energy to sustain rapid proliferation, migration, and survival. To meet these demands, they undergo distinct metabolic reprogramming.

According to the Warburg effect, cancer cells preferentially rely on glycolysis for energy production, rather than OXPHOS, even in the presence of adequate oxygen (“aerobic glycolysis”), allowing them to utilize glucose for both energy and macromolecule production [40,41]. Once cancer cells take up glucose, it is metabolized into pyruvate, which is mostly converted into lactate, generating an acidic tumor microenvironment. Despite the low efficiency in generating ATP, the glycolytic pathway provides metabolic intermediates used as building blocks for the biosynthesis of fundamental biomolecules such as DNA, proteins, and lipids [42,43]. However, despite the crucial role of the glycolytic pathway in cancer cell metabolism, its pharmacological inhibition has proven ineffective in arresting tumorigenesis. This ineffectiveness has been attributed to the remarkable metabolic plasticity of cancer cells, enabling them to undergo a metabolic rewiring and upregulate oxidative metabolism [44,45]. Additionally, evidence obtained in the last twenty years from molecular biology studies indicates that, despite highly active glycolysis, cancer cells also upregulate mitochondrial respiration to supply the energy and biosynthetic precursors necessary to sustain their anabolic growth [3,4,9,28,46,47,48]. Notably, the mitochondrial TCA/OXPHOS machinery has been found to be overactivated even in metastatic and drug-resistant cancer cells [49,50,51,52,53,54], as well as in the aggressive subpopulation of cancer stem cells (CSCs) [55,56,57,58,59], supporting their ability to evade drug-induced apoptosis and to maintain self-renewal and stemness characteristics.

The reprogramming of mitochondrial metabolism in cancer cells is frequently mediated by mutations in nuclear genes encoding metabolic enzymes, leading to the production of oncometabolites. For instance, mutations in genes involved in the TCA cycle, such as fumarate hydratase (FH), succinate dehydrogenase (SDH) (loss of function), and isocitrate dehydrogenases (IDH1/2) (gain of function) induce the accumulation of the oncometabolites fumarate, succinate, and 2-hydroxyglutarate (2-HG), respectively; these oncometabolites have been demonstrated to be deeply involved in the process of carcinogenesis by activating pleiotropic mechanisms, including epigenetic changes, stabilization of hypoxia-inducible factor-1α (HIF-1 α), and mitochondrial ROS generation [60]. Specifically, cancer cells carrying mutations in the IDH1/2 genes are characterized by a peculiar dependence on the OXPHOS program and sensitivity to OXPHOS inhibitors [61].

Altered expression of mitochondrial DNA (mtDNA) has been reported to occur in different tumors. Specifically, Bonekamp and coworkers demonstrated that the pharmacological inhibition of mitochondrial RNA polymerase leads to a reduced expression of subunits of the ETC complexes I, III, and IV of the OXPHOS machinery and is associated with a decrease in basal respiration and a significant antitumor effect in ovarian cancer cells [62]. Consistent with these observations, somatic mtDNA point mutations and gene copy alterations affecting these respiratory chain complexes have been observed in different types of tumor cells and found to correlate with increased mitochondrial biogenesis, ATP production, ROS generation, uncontrolled cell proliferation, and metastasis [47,63,64,65].

Remarkably, mutations in genes encoding TCA metabolic enzymes, as well as mtDNA mutations, have been widely reported to be associated with dysregulation of ROS and Ca^2+^ homeostasis, promoting tumorigenesis [66,67].

**Figure 1:**
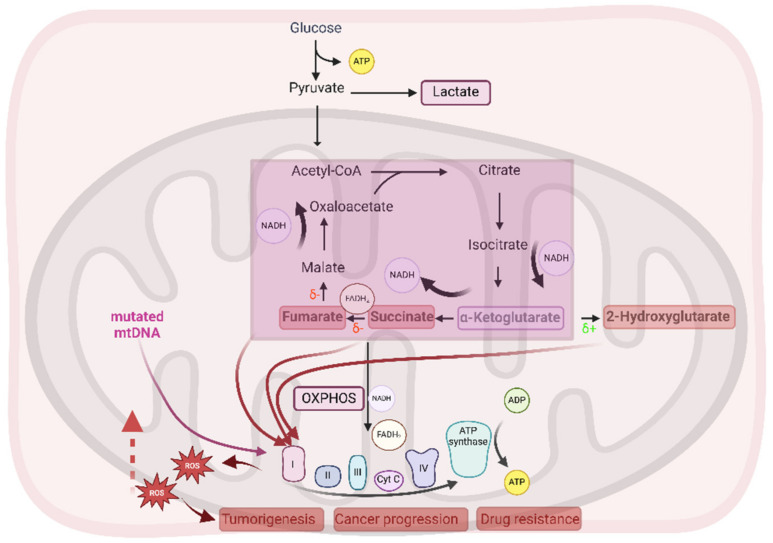
Schematic overview of the mitochondrial metabolic reprogramming occurring in cancer cells. Despite the presence of highly active glycolysis, functional mitochondrial respiration (OXPHOS) is present in cancer cells. Mitochondrial metabolic rewiring is frequently associated with mutations in nuclear genes encoding enzymes involved in the TCA cycle, such as succinate dehydrogenase and fumarate hydratase (δ−, loss of function) and isocitrate dehyadrogenase (δ+, gain of function), leading to the accumulation of the oncometabolites succinate, fumarate, and 2-hydroxyglutarate. Somatic mtDNA mutations affecting the expression of proteins of the ETC complexes have also been pointed out in tumor cells. Together, oncometabolites and mtDNA mutants promote dysregulation of the ETC activity, leading to ROS generation, accountable for tumor development and progression. OXPHOS—oxidative phosphorylation; TCA cycle—tricarboxylic acid cycle; mtDNA—mitochondrial DNA; ETC—electron transport chain. This figure was created with BioRender.com.

A schematic representation of mitochondrial metabolic reprogramming in cancer cells is illustrated in Figure 1.

Mitochondrial metabolic reprogramming extends beyond glucose metabolism to include amino acids and lipids. Through the glutaminolysis pathway, cancer cells convert glutamine into glutamate, fueling the TCA cycle and the formation of intermediates necessary for macromolecule biosynthesis to support anabolic growth. Moreover, tumor cells reprogram lipid metabolism, enhancing fatty acid uptake, promoting de novo lipogenesis, and altering fatty acid oxidation to support rapid growth, survival, and adaptation to the challenging conditions of the tumor microenvironment [28,47,48,68,69,70,71].

## 3. ROS Generation and Oxidative Stress

The family of ROS chemicals includes ions (superoxide anion, O_2_^−^), free radicals (hydroxyl radical, OH^•^), and neutral molecules (hydrogen peroxide, H_2_O_2_) [10,11]. Approximately 90% of cellular ROS are generated in mitochondria from the electron leakage of the ETC complexes I and III [72,73]. Under physiological conditions, mitochondrial ROS play important roles in different biological processes, including cell signaling, proliferation and death, vascular regulation, and immune responses [22]. On the other hand, it is now well recognized that dysregulated intracellular ROS levels and ROS homeostasis are deeply involved in the etiology of several diseases, including neurodegenerative disorders, cardiovascular diseases, and cancer [31,57,74,75,76].

**Figure 2:**
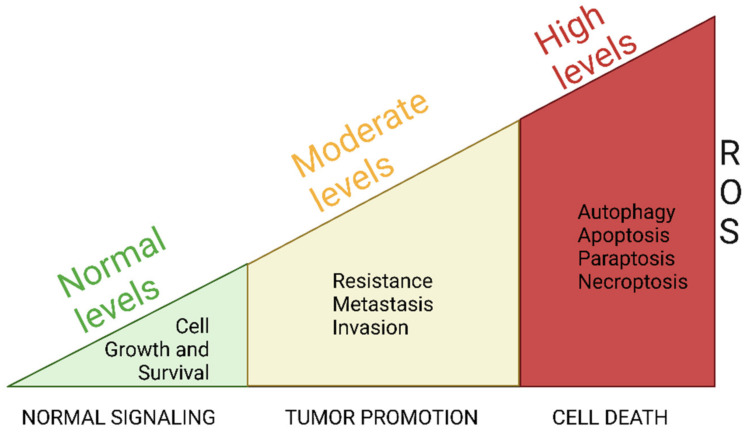
Differential effects of ROS levels in normal and cancer cells. In normal cells, low ROS levels are involved in the control of cell growth and survival. In cancer cells, moderate ROS levels are essential for cancer cells to sustain their proliferative, invasive, metastatic, and drug-resistant behavior. On the other hand, excessive ROS levels trigger oxidative stress, resulting in cancer cell death pathways. This figure was created with BioRender.com.

In normal cells, low ROS levels are essential for maintaining cell proliferation and survival [77]. In cancer cells, however, ROS play a dual role in regulating cell fate, promoting either proliferation or death. This duality has led to ROS being referred to as “rheostats” [78,79] or molecules that have “double-edged sword” properties [80,81]. A unique characteristic of cancer cells is, at least in part, responsible for this phenomenon: they can tolerate higher ROS levels than healthy cells. At moderate levels, ROS in cancer cells induce the transcription/activation of antioxidant enzymes, protecting them from ROS-induced oxidative damage. Additionally, moderate levels of ROS behave as mitogenic molecules for cancer cells, promoting mitogenic signaling pathways involved in cell proliferation, invasion, metastasis, and drug resistance. Only excessive intracellular ROS levels trigger oxidative stress, leading to the disruption of the redox signaling and the induction of oxidative damage (damage to mtDNA, proteins, and lipids), ultimately causing cell cycle arrest, cell death, and tumor suppression [75,81,82] (Figure 2).

In line with these observations, ROS have been proposed as potential markers for the prediction of response to anticancer therapies, and increasing their levels is a goal of anticancer strategies [77,83,84].

## 4. ROS-Associated Cell Death Pathways

As discussed above, excessive intracellular ROS levels promote oxidative stress that causes oxidative damage to proteins, DNA, and lipids to promote the deterioration of intracellular organelles, including mitochondria, as well as the plasma membrane, potentially resulting in cell death. Different programmed cell death pathways (autophagy, apoptosis, paraptosis, and necroptosis) have been reported to be involved in the pro-death effects of elevated ROS in cancer cells [79,81,85,86].

Autophagy is a degradation process that plays a pivotal role in the elimination of damaged intracellular molecules and organelles, previously engulfed by double-membrane vesicles called autophagosomes, which ultimately fuse with lysosomes, leading to the degradation and recycling of their molecular cargo [87]. An intricate bidirectional interplay exists between autophagy and ROS in cancer cells [88,89]. Specifically, under normal conditions, autophagy has been shown to remove intracellular ROS and ROS-generating organelles, thus reducing ROS levels, preventing ROS-induced cell death and promoting cell survival [90,91]. Conversely, in cancer cells, ROS can induce autophagy to promote either cell death or survival depending on their intracellular levels and the specific cell context [92]. The recent literature supports the involvement of the ROS/autophagic cell death axis in the activity of different synthetic and natural anticancer compounds [93]. In line with these observations, the depletion of autophagy-related proteins has been shown to increase cell proliferation [94,95,96,97]. Studies on ferroptosis, a form of programmed cell death, characterized by cell membrane rupture induced by iron-dependent accumulation of lipid peroxide, are consistent with these data. Ferroptosis is triggered by the autophagic pathway in cancer cells [98,99]. On the other hand, ROS-mediated autophagy has been reported to inhibit apoptosis and provide cytoprotective effects against oxidative stress-related cell death, contributing significantly to the development of drug resistance in various cancer cells [100,101].

Apoptosis is the most extensively studied form of cell death. It is characterized by two main pathways: the intrinsic mitochondrial pathway and the extrinsic pathway involving cell death receptors at the membrane level [102]. Intrinsic apoptosis occurs when an imbalance between anti-apoptotic proteins (Bcl-2 family proteins) and pro-apoptotic proteins (Bax, Bak, and Bok) is caused by intracellular events, leading to mitochondrial outer membrane permeabilization, cytochrome c release into the cytosol, and subsequent activation of executioner caspases (caspase-3 and -7) [103,104]. Extrinsic apoptosis pathways initiate when pro-death extracellular signals bind to death receptors (i.e., TNF or Fas) at the membrane level. Activation of death receptors induces the recruitment of adaptor proteins to form death-induced signaling complexes (DISCs) which, in turn, trigger the activation of a caspase cascade involving the initiator caspase-8 and, ultimately, the executioner caspases [105].

Elevated ROS levels are deeply involved in the activation of both the intrinsic and extrinsic apoptotic pathways by triggering cytochrome c release from the mitochondria and by activating death receptors, respectively [106,107].

Paraptosis is a type of programmed cell death characterized by extensive cytoplasmic vacuolation arising from endoplasmic reticulum (ER) and mitochondria swelling, while lacking the typical features of apoptosis [108,109]. Paraptosis is closely associated with ER stress, protein misfolding, alterations in Ca^2+^, and redox homeostasis [110]. An important component of this cell death mechanism is the role of the ER–mitochondria contact sites or MAMs (mitochondrial-associated ER membranes). Under severe stress, MAMs facilitate Ca^2+^ flux from ER to mitochondria, leading to mitochondrial dysfunction and excessive ROS production. The increase in ROS levels triggers paraptosis [110,111,112]. Thus, the interaction between dysregulated Ca^2+^ signaling and elevated ROS levels is central to the paraptotic cell death process [113].

Necroptosis, a caspase-independent necrosis-like cell death [114], is characterized by cell swelling, organelle shrinkage, chromatin condensation, nuclear membrane dilation, and loss of plasma membrane integrity [115,116]. Necroptosis, activated by stressful stimuli including tumor necrosis factor (TNF), chemotherapy, or hypoxia [117], involves receptor-interacting serine/threonine kinase 1 (RIPK1)-driven activation of RIPK3, which activates mixed lineage kinase domain-like pseudokinase (MLKL). MLKL promotes pore formation, loss of membrane integrity, and release, resulting in inflammation and cell death [116,118]. Accumulating evidence suggests that mitochondrial ROS play a key role in driving necroptosis in cancer cells [119,120]. For instance, ROS generation has been shown to contribute to necroptosis induced by the natural compound emodin in renal cancer cells [121] and to be involved in MLKL activation in lung cancer cells [122]. RETRA is a small molecule identified for its ability to bind the mutant p53/p73 complex, enabling p73 to activate a set of p53-regulated genes that trigger cell death [123]. Interestingly, Mohanty and coworkers reported that RETRA activity is independent of p53 status, since it induces necroptosis in both p53 mutant and wild-type cervical cancer cells, through ROS-mediated activation of RIPK1, RIPK3, and MLKL [124].

## 5. Tocotrienols

As pointed out above, in addition to an increased glycolytic pathway, mitochondrial metabolic reprogramming is active in cancer cells, providing them with the high energy and biosynthetic precursors necessary to support their growth and survival. Moreover, mitochondria are the major intracellular producers of ROS through electron leakage of the ETC complexes, and excessive levels of ROS above the cytotoxic threshold have been shown to impair redox homeostasis, triggering different types of cancer cell death. Based on these observations, it has become increasingly recognized that mitochondrial metabolism-targeting and ROS-stimulating compounds, both synthetic and natural, might represent effective anticancer therapeutic interventions [21,22,23,24,25,26,27,28,29,30,31,125].

**Figure 3:**
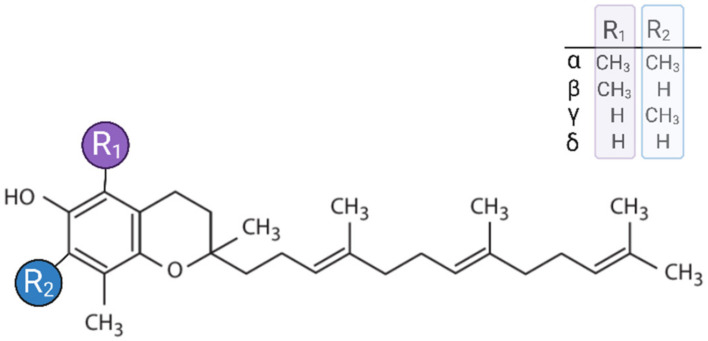
Chemical structure of the four tocotrienol isoforms (α, β, γ, and δ). This figure was created with BioRender.com.

The vitamin E family exists in two groups of compounds: α-, β-, δ-, and γ-tocopherols (TPs) and the corresponding tocotrienols (TTs). Structurally, they are composed of a chromanol nucleus linked to a 15-carbon isoprenoid side chain that is saturated in TPs and unsaturated in TTs. The various isoforms of TPs and TTs are distinguished by the number and position of the methyl groups on the chromanol ring: the α-isoform contains three methyl groups, whereas the β- and γ- have two, and the δ-form only has one methyl group (Figure 3).

Both TPs and TTs are absorbed in the lumen of the small intestine and taken up into liver cells through the α-TTP (α-TP transport protein) transporter. From the liver, they are secreted into circulating lipoproteins, allowing their delivery through the bloodstream to target organs. TTs display a lower affinity for α-TTP compared to TPs; based on this, their actual bioavailability has been questioned. Despite these concerns, preclinical and clinical studies demonstrated that TTs are detectable in blood and tissues after oral administration, suggesting that they may reach their target tissues through alternative pathways [126,127,128,129]. Based on their lipophilic properties, the bioavailability of TTs after oral administration has been widely questioned. It has been demonstrated that taking TTs with food enhances the amount of these compounds absorbed at the intestinal level; specifically, fatty meals increase TT solubility thanks to the formation of micelles that enhance the area of absorption at the intestinal level caused by the secretion of the digestive enzyme pancreatic lipase and the stimulation of bile salts [130]. Recently, to increase TT bioavailability after oral administration, novel formulations of these compounds have been developed and are currently being investigated. These include self-emulsifying drug delivery systems and nanoformulations such as nanoparticles, nanoemulsions, and solid lipid nanoparticles [130,131]. Moreover, alternative routes of administration (transdermal application of TT-containing gels, subcutaneous injection of TT-containing nanodroplets, and sublingual delivery of TT-based tablets) have been recently proposed [132].

TTs can be purified from a variety of plant sources, including annatto seeds, palm oil, and rice bran [133,134]. They offer significant health benefits, particularly in the management and prevention of chronic diseases thanks to their neuroprotective, cholesterol lowering, anti-diabetic, and anti-osteoporotic properties, which are not displayed by TPs [135,136,137,138]. Specifically, we demonstrated that, in murine MC3T3-E1 osteoblastic cells, δ-TT prevents the effects of t-BHP (tert-butylhydroperoxide) on cell viability and apoptosis. This protective effect is primarily due to a reduction in intracellular ROS levels and an increase in the GSH/GSSG (glutathione/glutathione disulfide) ratio, a key indicator of cellular redox state [139]. This suggest enhanced activation of antioxidant defense systems [140].

In recent decades, δ-TT and γ-TT have garnered significant interest as antitumor compounds based on their ability to promote cell death and to counteract the migratory, invasive, and proangiogenic properties of different types of cancer cells [34,35,36,37,38,39,141,142,143,144].

In the following sections, we address the molecular bases underlying the anticancer properties of TTs, with a specific focus on the recent progress in understanding the involvement of mitochondrial metabolic reprogramming and ROS generation.

### Anticancer Activity

Over the past two decades, many in vitro and in vivo investigations have pointed out that TTs, but not TPs, are endowed with significant anticancer properties against different types of tumors [36,37,141,144,145,146], such as melanoma [36], prostate cancer [143], colon cancer [147,148], and breast cancer [149]. Specifically, δ- and γ-TT have been pointed out as the most active TT isoforms [150,151] in impairing tumor cell growth, progression, and stemness, as well as in promoting cell death pathways through multiple molecular mechanisms [35,141,152,153,154,155]. Notably, in our laboratory, we could demonstrate that δ-TT significantly impairs prostate cancer cell viability, while sparing normal prostate epithelial cells [156]. In different types of cancer cells (breast, prostate, lung, pancreatic, gastric, colon cancer cells, etc.), TTs have been reported to induce cell cycle arrest through the modulation of the expression of cell cycle-related proteins, such as cyclins, cyclin-dependent kinases (CDKs), and the cell cycle inhibitors p21 and p27 [157,158,159,160,161]. Moreover, different signaling pathways, including PI3K/Akt/mTOR, MEK/ERK, transforming growth factor β (TGFβ), epidermal growth factor receptors (ErbBs), receptor signal transducer and activator of transcription (STAT), and nuclear factor-κB (NFκB), have been shown to mediate TTs-induced cell growth arrest or even apoptosis in tumor cells [35,37,154,158,162,163,164,165,166,167,168]. Importantly, TTs were recently shown to trigger an anticancer immune response by increasing the activation/recruitment of cytotoxic T-lymphocytes while reducing suppressive immune cell infiltration [169].

TTs were also reported to induce both the intrinsic and extrinsic apoptosis pathways in cancer cells through the modulation of different apoptosis-related proteins, such as Bax, Bcl-2, Fas, Fas ligand, Trail, and caspase-8 [170]. However, the involvement of the pro-apoptotic protein p53 in their activity is still a matter of debate. For instance, in breast cancer cells, δ- and γ-TT trigger apoptosis via the mitochondrial pathway and the upregulation of the growth arrest marker p53 [171]; conversely, β-TT-induced apoptosis was reported to occur through a p53-independent apoptosis pathway in the same type of cancer cells [167].

As discussed above, the ER is a complex and dynamic organelle where protein synthesis, their folding, and Ca^2+^ homeostasis are strictly controlled. Different conditions, both extrinsic and intrinsic, can trigger ER stress-unfolded protein response (UPR), leading to the accumulation of unfolded/misfolded proteins and to Ca^2+^ homeostasis deregulation [172]. The involvement of ER stress in the pro-apoptotic activity of several synthetic and natural anticancer compounds is now well recognized [38,112,173,174,175]. Specifically, TTs were shown to trigger the intrinsic apoptosis process through the activation of ER stress-related pathways (i.e., BIP/PERK/p-eIF2α/ATF4/CHOP, IREα, and caspase-4) in melanoma, colorectal, cervical, and breast cancer cells [38,112,161,176,177]. Potent phytochemical compounds, including TTs, promote apoptosis in tumor cells through the activation of the ER stress–autophagic pathway [178,179]. Taking advantage of a transcriptomic analysis, Pang and coworkers demonstrated that, in chondrosarcoma cells, δ- and γ-TT induce a cell cycle arrest followed by ER stress-mediated autophagy and apoptosis [180].

Paraptosis has also been demonstrated to be involved in the anticancer activity of TTs. In our laboratory, we pointed out that δ-TT induces apoptosis and paraptosis, characterized by intense cytoplasmic vacuolation, in castration-resistant prostate cancer (CRPC) and melanoma cells [156,181]. In line with these observations, Zhang and coworkers demonstrated that, in colon cancer cells, γ-TT induces a paraptosis-like cell death mediated by a downregulation of the Wnt signaling pathway [182].

Moreover, Montagnani Marelli et al. recently reported that δ-TT triggers apoptosis and necroptosis in prostate cancer cells while overcoming docetaxel resistance [155].

TTs are also endowed with peculiar antimetastatic and antiangiogenic activities through the regulation of cancer cell motility, extracellular matrix remodeling, and vessel formation [183]. In vitro and in vivo studies demonstrated that, in pancreatic ductal adenocarcinoma cells, δ-TT impairs migration, invasion, and the expression of biomarkers of the epithelial to mesenchymal transition (EMT), a process which fosters the metastatic ability of cancer cells [184]. In this context, in highly invasive breast cancer cells, γ-TT reversed the EMT process through the upregulation of epithelial markers (E-cadherin) and the suppression of mesenchymal markers (vimentin, β-catenin, and fibronectin) [185]. In lung cancer cells and in human gastric adenocarcinoma cells, δ-TT and γ-TT downregulated the expression of metastatic markers, such as matrix metalloproteinase-2 and -9 (MMP-2 and MMP-9) and urokinase-type plasminogen activator (uPA), which are deeply involved in the degradation of the extracellular matrix, while increasing the expression and activity of tissue inhibitors of metalloproteinases (TIMP-1 and TIMP-2) [164,186,187]. In this context, it has been pointed out that, in mouse and human mammary cancer cells, γ-TT affects the expression of proteins deeply involved in cytoskeleton organization (Rac1, WAVE2, and Arp2), resulting in a significant decrease in cell motility and invasive behavior [188]. Similar antimetastatic activities of γ-TT were observed in prostate and melanoma cancer cells [189,190].

Another key event for metastasis formation, angiogenesis, the growth of new vessels from pre-existing ones, represents an effective target of the anticancer activity of TTs. In vitro and in vivo studies demonstrated that TTs impair the proangiogenic properties of cancer cells by reducing the expression of vascular endothelial growth factor (VEGF), the essential mitogenic factor for vascular endothelial cells and the key driver of angiogenesis [184,191,192]. Consistently, δ-TT and γ-TT were reported to suppress hypoxia-induced VEGF secretion from different types of cancer cells (prostate, colorectal, hepatocellular, and gastric cancer cells) [193,194,195]. In addition, TTs also displayed antiangiogenic properties by directly targeting endothelial cells. Specifically, in vitro and in vivo studies pointed out that TTs inhibit VEGF-induced endothelial cell proliferation, migration, and tube formation ability [196,197].

The positive outcomes of TT treatment obtained from in vitro and preclinical studies promoted some clinical trials to investigate the safety and efficacy of TTs in cancer patients. Oral administration of δ-TT in preoperative pancreatic ductal neoplasia patients was found to reach bioactive levels in the blood, to be free of side effects, and to induce apoptosis in tumor cells [198]. Moreover, a combination treatment of δ-TT and the antiangiogenic drug bevacizumab in patients diagnosed with stage III, multidrug-resistant ovarian cancer pointed out a prolonged progression-free survival and lifespan together with the absence of side effects [199]. However, contrasting results have been recently reported in breast cancer patients treated with δ-TT in combination with chemotherapeutic drugs [200].

Interestingly, while several other natural anticancer drug candidates, such as quercetin, sulforaphane, berberine, luteolin, α-lipoic acid, curcumin, ginsenoside, silibinin, and carnosol, are under investigation, TTs are recognized as potentially superior starting points for novel drug development. This is also reflected by the significantly higher number of clinical trials evaluating their safety, bioavailability, disease-preventive properties, and therapeutic efficacy in cancer patients (https://clinicaltrials.gov/).

## 6. Targeting Mitochondrial Metabolic Reprogramming and ROS Generation

Based on their crucial roles in cancer cell survival and proliferation, mitochondrial metabolic reprogramming and ROS generation are currently considered attractive targets for novel anticancer drugs, including natural compounds.

**Figure 4:**
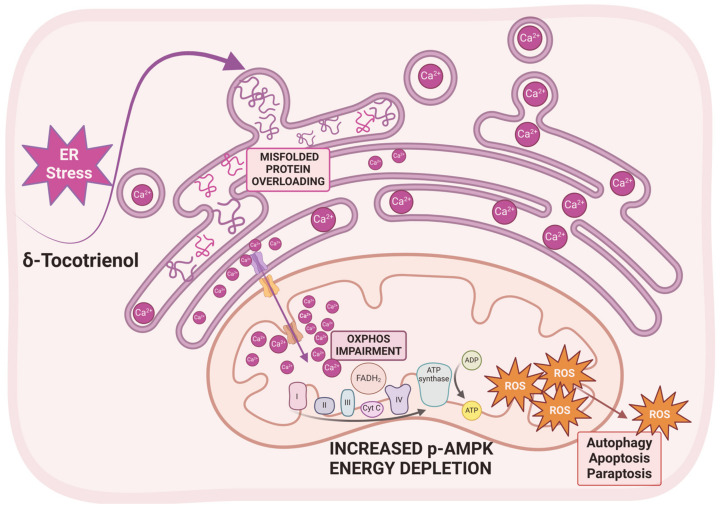
Schematic representation depicting the involvement of mitochondrial metabolic reprogramming and ROS generation in the anticancer activity of δ-TT (δ-Tocotrienol). δ-TT triggers ER stress, leading to Ca^2+^ release from ER and its accumulation in mitochondria. Ca^2+^ overload promotes an impairment of mitochondrial metabolic functions associated with ROS overgeneration, ultimately responsible for the induction of cancer cell death pathways. ER—endoplasmic reticulum; OXPHOS—oxidative phosphorylation; p-AMPK—phosphorylated adenosine monophosphate-activated protein kinase. This figure was created with BioRender.com.

In our laboratory, we pointed out that δ-TT triggers ER stress-mediated intrinsic apoptosis and paraptosis in melanoma and in CRPC cells [112,156,177,181]. Mechanistically, we demonstrated that, in human melanoma cells, δ-TT promotes the paraptotic cell death program, characterized by ER/mitochondrial dilation and cytoplasmic vacuolation. Specifically, we pointed out that δ-TT triggers Ca^2+^ release from the ER and its accumulation in mitochondria, through Ca^2+^ channels located at the MAM level, leading to an impairment of mitochondrial metabolic functions, such as reduced expression of proteins of the OXPHOS complex I, marked decrease in O_2_ consumption and mitochondrial membrane potential (∆Ψm), an index of mitochondrial dysfunction), reduced ATP production, increased phosphorylation of the energy sensor AMPK, and ROS overgeneration, ultimately responsible for the induction of paraptotic cell death [181]. Notably, the inhibition of Ca^2+^ release from ER and its accumulation in mitochondria prevents mitochondrial ROS generation and paraptosis, highlighting the key role of the Ca^2+^/ROS axis in δ-TT-induced paraptosis. In accordance with these observations, we showed that, in CRPC cells, δ-TT impairs mitochondrial respiration by decreasing O_2_ consumption and ATP generation through the downregulation of the expression of ETC proteins (complex I, II, and IV) and the mitochondrial ∆Ψm. δ-TT also triggers mitochondrial Ca^2+^ overload and ROS overproduction, which are deeply involved in its anticancer activities (autophagy and mitophagy, apoptosis, and paraptosis) [201] (Figure 4).

Our data are consistent with those of Viola and coworkers, which reported that, in HER2/Neu-overexpressing breast cancer cells, δ-TT induces mitochondrial destabilization and impairment of ATP production, associated with alterations in stress/survival signaling pathways (p38 and ERK1/2), and increased ROS production leading to apoptotic cell death [202].

Similar observations were reported for the γ-TT isoform of tocotrienols in human gastric adenocarcinoma cells, where it was demonstrated to reduce the expression levels of mitochondrial ETC complex I and II proteins, ATP production, and ∆Ψm. Moreover, this mitochondrial functional impairment was found to be strictly related to ROS overproduction, which is responsible for cancer cell apoptotic death. Notably, γ-TT-treated cancer cells were also found to activate the glycolytic pathway to compensate for the defective OXPHOS process; however, this metabolic switch was not sufficient to sustain the ATP generation rate in these cells [203]. Taking advantage of the RNA-seq analysis, Xie and Yan demonstrated that, in human gastric cancer cells, γ-TT treatment triggers apoptosis by inhibiting the mitochondrial protein-containing complexes, including the NADH–dehydrogenase complex I, and the OXPHOS pathway through downregulating the expression levels of Notch1 and Notch2 [204].

Alterations in ROS homeostasis have also been shown to be involved in the anticancer activity of TTs. We demonstrated that δ-TT triggers JNK- and p38-dependent apoptosis by promoting an overall cellular ROS production as well as a significant increase in mitochondrial ROS generation in ovarian cancer cells; notably, ROS removal by pretreatment with the specific scavenger NAC (N-acetyl cysteine) significantly counteracted the antitumor activity of δ-TT [168]. Wilankar et al. showed that γ-TT inhibits proliferation and induces extrinsic and intrinsic apoptosis in human T cell lymphoma Jurkat cells in a dose-dependent manner. Mechanistic investigations revealed that γ-TT treatment results in increased mitochondrial ROS production, Ca^2+^ release, activation of JNK, and suppression of ERK and p38 MAPK activity. γ-TT also triggers intrinsic apoptosis by inducing cytochrome c release from the mitochondria [170]. Montagnani Marelli and coworkers reported that δ-TT exerts antiproliferative and pro-apoptotic activities in human hepatocarcinoma cells, triggering ROS release from mitochondria associated with their functional dysregulation, resulting in the decrease of ∆Ψm. Structurally, δ-TT promotes mitochondrial fission, followed by the autophagic removal of damaged organelles (mitophagy) [205]. Interestingly, in these cells, TTs display the ability to enhance the antiproliferative, pro-apoptotic, and antimetastatic effects of other molecules; i.e., δ-TT potentiates the effect of IFN-α (interferon-α) by promoting ROS generation and inducing alterations in the Notch1 and ERK signaling pathways [206]. Analogously, γ-TT potentiates the caspase-independent apoptosis of 6-gingerol in colorectal cancer cells; this synergistic effect was mediated by the activation of the ER stress pathway, closely related to ROS production and oxidative stress, and mitochondrial dysfunction [161]. Finally, an optimized W/O/W (water-in-oil-in-water) nanoemulsion formulation encapsulating a tocotrienol-rich fraction, (TRF; TTs extracted from palm oil, consisting of γ-, α-, and δ-TT) and caffeic acid with cisplatin (CIS) was developed, and its effects on lung and hepatocellular carcinoma cell viability were investigated. It was observed that a combination of TRF and caffeic acid with CIS synergistically induces ROS overproduction correlated with cancer cell apoptosis and reduces the doses of CIS required for cell death induction with a consequent decrease in cytotoxicity and side effects of the therapeutic approach [207].

Together, these data strongly support that mitochondrial metabolic reprogramming and ROS generation represent effective molecular targets mediating the anticancer activity of TTs (Table 1).

## 7. Conclusions

Mitochondrial metabolic reprogramming and ROS generation are key hallmarks of cancer and appealing molecular targets for novel anticancer drugs, including natural compounds, such as tocotrienols (TTs). Specifically, the δ- and γ-TT isoforms have shown promising anticancer properties by promoting cancer cell death. Recent studies have pointed out that δ- and γ-TT significantly revert mitochondrial functional dynamics by decreasing O_2_ consumption, ATP production, and OXPHOS activity, while triggering mitochondrial ROS generation, leading to oxidative damage and cell death. These mechanisms underlie the anticancer properties of TTs.

While TTs may be considered promising preventive and therapeutic agents against cancer, challenges remain, particularly their low absorption level in the body due to being fat-soluble compounds. Novel TT formulations (self-emulsifying drug delivery systems, nanoformulations such as nanovesicles, nanoemulsions, and solid lipid nanoparticles) have been developed and tested in preclinical studies. However, clinical studies are warranted to confirm the therapeutic potential of TTs, either alone or in combination with standard treatments, to definitely assess their potential as an effective approach for cancer care.

## Figures and Tables

**Table 1:** Involvement of mitochondrial metabolic reprogramming and ROS generation in the pro-death activity of TTs in human cancer cells.

Tocotrienol (TT)	Target Cells	Biological Effects	Cell Death Pathways	Ref.
δ-TT	Melanoma cells	Impairment of mitochondrial respiration (↓ O_2_ consumption and ATP production, downregulation of ETC complex I proteins, ↓ mitochondrial membrane potential, AMPK activation), mitochondrial overload of ER-derived Ca^2+^ and ROS generation	Paraptosis	[181]
δ-TT	Castration-resistant prostate cancer (CRPC) cells	Impairment of mitochondrial respiration (↓ O_2_ consumption and ATP production, downregulation of ETC complex I, II, and IV proteins, ↓ mitochondrial membrane potential), intracellular and mitochondrial Ca^2+^ overload and ROS overproduction	Mitophagy-related apoptosis and paraptosis	[201]
δ-TT	HER2/Neu-overexpressing breast cancer cells	Mitochondrial destabilization, impairment of ATP production, alterations in stress/survival signaling pathways (p38 and ERK1/2), ↑ ROS production	Apoptosis	[202]
γ-TT	Gastric cancer cells	Impairment of mitochondrial respiration (↓ O_2_ consumption, ATP depletion, ↓ expression levels of ETC complex I and II subunits, ↓ mitochondrial membrane potential), ↑ intracellular ROS levels	Apoptosis	[203]
γ-TT	Gastric cancer cells	Inhibition of ETC complex I (NADH–dehydrogenase complex); impairment of the OXPHOS pathway, downregulation of notch 1 and 2 gene expression	Apoptosis	[204]
δ-TT	Ovarian cancer cells	Intracellular and mitochondrial ROS overproduction and downstream JNK/p38 activation	Apoptosis	[168]
γ-TT	T cell lymphoma cells	Mitochondrial ROS overproduction, Ca^2+^ release, activation of JNK and suppression of ERK and p38 MAPK pathways	Extrinsic and intrinsic apoptosis	[170]
δ-TT	Hepatocellular carcinoma cells	↑ mitochondrial ROS release associated with mitochondrial fission	Autophagy/mitophagy-dependent apoptosis	[205]
δ-TT + IFN-α	Hepatocellular carcinoma cells	↑ ROS generation and alterations of Notch1 and ERK signaling pathways	Apoptosis	[206]
γ-TT + 6-gingerol	Colorectal cancer cells	Activation of the ER stress pathway, closely related to ROS production and oxidative stress	Caspase- Independent apoptosis	[161]
Nanoemulsion formulation encapsulating TRF (tocotrienol-rich fraction and caffeic acid with cisplatin)	Lung and hepatocellular carcinoma cells	↑ intracellular ROS levels	Apoptosis	[207]
